# Paradoxical Splenic Signal Loss on Chemical-Shift MRI in Acute Toxic Hepatitis After Occupational Petroleum Sludge Exposure: A Case Series

**DOI:** 10.3390/diagnostics16213704

**Published:** 2026-11-09

**Authors:** Bişar Akbaş, Mustafa Oğuz Tuğcan, Fatma Efe, Nisanur Yardımcı, Ferhat Can Pişkin, Eylül Akbal, Hasan Bilen Onan

**Affiliations:** 1 Department of Radiology, Faculty of Medicine, Çukurova University, 01330 Adana, Türkiye; efefatma696@gmail.com (F.E.); ferhatcpiskin@gmail.com (F.C.P.); eylulakbal@hotmail.com (E.A.); 2 Department of Emergency Medicine, Faculty of Medicine, Çukurova University, 01330 Adana, Türkiye; oguztugcan@gmail.com (M.O.T.); nsnralz@gmail.com (N.Y.); 3 Neuroradiology Private Practice, 01060 Adana, Türkiye; bilenonan@hotmail.com

**Keywords:** occupational liver disease, solvent-induced hepatotoxicity, chemical-shift MRI, paradoxical splenic signal loss, petroleum sludge

## Abstract

**Background:** Acute toxic hepatitis after work-related exposure to petroleum products is rare. Its imaging features are not well known. **Methods:** Seven men aged 18–35 years cleaned the same petroleum storage tank for two days. Symptoms began about 5 days later. Ultrasound and CT were done at admission. Non-contrast 3 T MRI with in-phase and opposed-phase imaging was done about 10 days after exposure. The splenic signal intensity ratio (SIR, in-phase/opposed-phase) was compared with that of 15 age- and sex-matched healthy controls. **Results:** At admission, median ALT was 481 U/L, median AST 227 U/L, and median total bilirubin 6.1 mg/dL. The injury was hepatocellular by R ratio in the six patients with raised ALT. Periportal edema was seen in 6 of 7 patients. No patient had biliary obstruction or vascular thrombosis. Notably, all seven patients demonstrated paradoxical splenic signal loss on in-phase compared with opposed-phase MRI, with a median splenic signal intensity ratio (SIR) of 0.77 (IQR, 0.74–0.81; range, 0.70–0.87). Splenic SIR was lower in patients than in healthy controls (median 0.77 vs. 1.05; *p* < 0.001). All patients recovered with supportive care. **Conclusions:** In this single exposure cluster, the spleen lost signal on in-phase images. This pattern is usually linked to iron or other T2* effects. The finding is preliminary. It needs confirmation with iron measurement, hemolysis tests, and follow-up MRI.

## 1. Introduction

Petroleum products and organic solvents, such as benzene, toluene, and xylene, can injure the liver. Cases have been reported after work in refineries, painting, printing, and tank cleaning [1,2,3]. Most reports describe single patients or small groups [3,4]. A recent cohort study found that ALT rose more over time in workers exposed to benzene, toluene, ethylbenzene, and xylene (BTEX) than in unexposed workers [5]. Environmental chemicals can also cause fatty liver and toxicant-associated steatohepatitis [6]. Pérez et al. studied 95 refinery workers exposed to aromatic hydrocarbons. Transaminases were raised in 29% of them, and about half of these workers had fatty livers on ultrasound [7]. Teschke reviewed 60 patients with liver injury from aliphatic halogenated hydrocarbons [8]. These studies give clinical and laboratory data, but little imaging data.

In toxic liver injury, ultrasound, CT, and MRI findings are usually nonspecific [3,9]. Imaging mainly helps to rule out other causes, such as biliary obstruction or vascular disease. The diagnosis still depends on the exposure history and laboratory tests [1,3,10,11].

Dual-echo chemical-shift MRI collects in-phase and opposed-phase gradient-echo images in one breath-hold. It is mostly used to detect fat in the liver [12,13,14]. It can also show iron. Iron shortens T2*, so tissue that contains iron loses more signal at a longer echo time. In most dual-echo sequences, the in-phase echo comes second. Iron-containing tissue therefore looks darker on in-phase images [13,15,16]. This is the opposite of the pattern seen with fat. In the spleen, it usually points to iron stored in macrophages, for example after transfusion or hemolysis [15,16]. Multi-echo R2* mapping is now the preferred method to measure tissue iron [17,18,19].

To our knowledge, this pattern has not been described in hydrocarbon-related toxic hepatitis. We saw it in all seven workers of one exposure cluster. Here, we describe the clinical, laboratory, and imaging findings of these workers. We also compare their splenic signal ratio with that of healthy controls. We present the splenic finding as a preliminary observation, not as a validated diagnostic sign.

## 2. Materials and Methods

### 2.1. Study Design and Patients

This retrospective case series was done at Çukurova University Faculty of Medicine, Adana, Türkiye. We included seven men with acute toxic hepatitis after the same occupational exposure to petroleum sludge. We used four inclusion criteria: (1) documented exposure to petroleum sludge; (2) symptoms of acute hepatitis (for example epigastric pain, loss of appetite, nausea, or jaundice) within one week of exposure; (3) raised ALT, AST, or bilirubin during the stay; and (4) no other cause of liver injury (Section 2.4). None of the patients had known liver disease, alcohol use, or use of hepatotoxic drugs, herbal products, or other chemicals.

The study was approved by the Çukurova University Faculty of Medicine Research Ethics Committee (approval no. 159, dated 3 October 2025) and was conducted in accordance with the Declaration of Helsinki. Written informed consent was obtained from all seven patients. The requirement for informed consent was waived for the healthy control group due to the retrospective use of anonymized MRI data.

### 2.2. Exposure

All seven workers were exposed during the same work period. They cleaned a large petroleum storage tank together. The work lasted 2 days, and the mean exposure time was about 10 h per worker. Because they worked side by side, their exposure was similar. The exposure was probably by inhalation and skin contact. The sludge was not analyzed, and no air measurements were made. Petroleum sludge is a mixture and a hazardous waste [20]. It usually contains aliphatic and aromatic hydrocarbons, polycyclic aromatic hydrocarbons, water, solids, and sometimes heavy metals [21,22]. We therefore cannot link the injury to a single chemical.

Symptoms began about 5 days after exposure, and the men came to our emergency department soon after. MRI was done on hospital day 5, about 10 days after exposure.

### 2.3. Clinical Evaluation and Treatment

At admission, patients reported epigastric pain, fatigue, and loss of appetite. Nausea, vomiting, changes in bowel habits, and low-grade fever were also common. All patients reported itching. All had epigastric tenderness without signs of peritonitis, and four had visible jaundice. Mental status was checked every day, and hepatic encephalopathy was graded with the West Haven criteria. No patient developed confusion or asterixis.

All patients were treated in the intensive care unit. Treatment was supportive. It included intravenous N-acetylcysteine [23] and oral ursodeoxycholic acid. We recorded the length of stay and any complications.

### 2.4. Laboratory Tests

Other causes of liver injury were excluded. Tests for viral hepatitis and autoimmune liver disease were negative in all patients. Salicylate and digoxin levels and pseudocholinesterase activity were normal. Doppler ultrasound excluded biliary obstruction and vascular thrombosis.

Laboratory tests were repeated every day during the stay. They included AST, ALT, alkaline phosphatase (ALP), gamma-glutamyl transferase (GGT), total and direct bilirubin, lactate dehydrogenase (LDH), albumin, INR, prothrombin time, creatinine, blood urea nitrogen, lactate, creatine kinase, D-dimer, and blood gases. Indirect bilirubin was total minus direct bilirubin. We used the R ratio to classify the injury: R = (ALT/ULN) ÷ (ALP/ULN). R ≥ 5 was hepatocellular, R ≤ 2 cholestatic, and R between 2 and 5 mixed [24,25,26]. The upper limits of normal (ULN) in our laboratory were 41 U/L for ALT and 129 U/L for ALP. MELD-Na was calculated at admission.

We used LDH and indirect bilirubin as indirect markers of hemolysis. Haptoglobin, reticulocyte count, blood smear, ferritin, serum iron, and transferrin saturation were not measured. Therefore, the presence or absence of hemolytic anemia could not be confirmed in any patient.

### 2.5. Imaging Protocol

All patients had abdominal ultrasound and non-contrast CT at admission. Hepatomegaly was defined as a liver length of ≥16 cm in the right midclavicular line [27]. Gallbladder wall thickening was defined as a wall thickness above 3 mm [28]. We also recorded periportal edema, liver echotexture and attenuation, ascites, and enlarged lymph nodes at the porta hepatis or celiac axis.

On hospital day 5, all patients had non-contrast abdominal MRI on a 3.0 T GE scanner (SIGNA, Milwaukee, WI, USA) with a 16-channel phased-array body coil. The protocol included a breath-hold axial dual-echo T1-weighted gradient-echo sequence, an axial fat-suppressed T2-weighted sequence, and diffusion-weighted imaging (DWI) with ADC maps. In the dual-echo sequence, the opposed-phase echo (TE 1.18 ms) came first, and the in-phase echo (TE 2.35 ms) came second. TR was 111 ms. Other parameters, including slice thickness, slice gap, field of view, matrix, and acquisition time, are given in Table 1.

### 2.6. Image Analysis

One abdominal radiologist with 7 years of experience reviewed all MRI examinations. The reader was not blinded. He knew which examinations belonged to patients and had access to the clinical history and laboratory results. Control examinations were measured in the same way.

Three circular regions of interest (ROIs) of about 50 mm^2^ were placed in the splenic parenchyma on the in-phase image. ROIs were kept away from the hilum, large vessels, splenic edges, and visible artifacts. The same ROIs were copied to the same position on the opposed-phase image of the same slice. We used the mean of the three ROIs. SIR was calculated as SI(in-phase)/SI(opposed-phase).

We used SIR < 0.90 (more than 10% signal loss on the in-phase image) to describe the findings. We chose this value after the measurements were made (post hoc). It is not based on earlier studies and has not been validated. The main analysis therefore used SIR as a continuous variable.

### 2.7. Control Group

The control group consisted of 15 healthy male adults matched to the patients by sex and age (median age, 26 years; range, 19–34 years). None had a known systemic disease or a history of liver disease, hemolytic disorders, iron overload, blood transfusion, or viral hepatitis. Their liver function tests and abdominal MRI were normal. All controls were examined on the same 3.0 T scanner with the same dual-echo T1-weighted sequence and parameters Table 2. Because the controls had no liver disease, matching for the severity of liver injury or for the timing of MRI relative to symptom onset was not applicable.

### 2.8. Statistical Analysis

Continuous variables are shown as median, interquartile range (IQR), and range. Categorical variables are shown as counts and percentages. We compared splenic SIR between patients and controls with the two-sided exact Mann–Whitney U test. A *p* value below 0.05 was considered significant. The sample was small, and all patients came from one exposure event, so the observations may not be independent. The *p* value should therefore be read as exploratory. Laboratory values were plotted over time for each patient. We used Python 3 (SciPy, Matplotlib) and Microsoft Excel.

## 3. Results

### 3.1. Patients

The seven men had a median age of 26 years (IQR 22.5–27.5; mean 25.6; range 18–35). Symptoms started about 5 days after exposure. MRI was done on hospital day 5, about 10 days after exposure. No patient met the criteria for acute liver failure. None had encephalopathy, and only one patient had an INR above 1.5 (1.70). All were hemodynamically stable at admission.

### 3.2. Laboratory Findings

Admission results are shown in Table 1. Median ALT was 481 U/L (IQR 351.5–575; range 36–668), and median AST was 227 U/L (IQR 160–315.5; range 24–345). ALT was higher than AST in six patients. The median AST/ALT ratio was 0.34 (range 0.28–1.01). In one patient (case 5), the admission ALT was recorded as 8 U/L, and it was 361 U/L the next day. We took the first value as a probable recording error and used the day-2 value. Median ALP was 128 U/L (range 48–172), so ALP was normal or only slightly raised. GGT was clearly raised (median 317 U/L; range 25–624).

**Figure 1:**
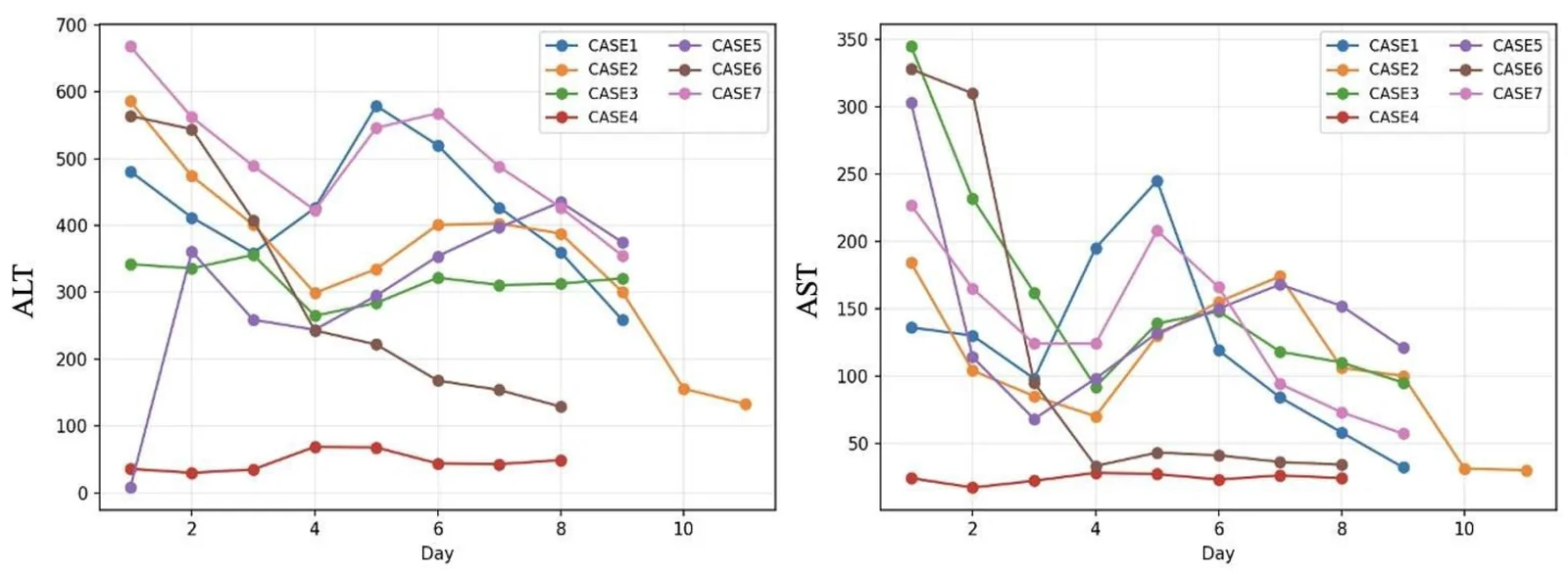
Serum ALT (**left**) and AST (**right**) from admission to discharge in the seven patients. Day 1 is the day of admission.

The R ratio was above 5 in all six patients with raised ALT (median 12.7; range 10.7–22.4). The pattern was therefore hepatocellular. Raised GGT and direct bilirubin showed a cholestatic component as well. One patient (case 4) had only mild changes. His ALT peaked at 69 U/L on day 4, and his bilirubin, ALP, and GGT stayed normal. We kept him in the series because he had the same exposure and symptoms as the others. Figure 1 shows the course of the transaminases.

**Figure 2:**
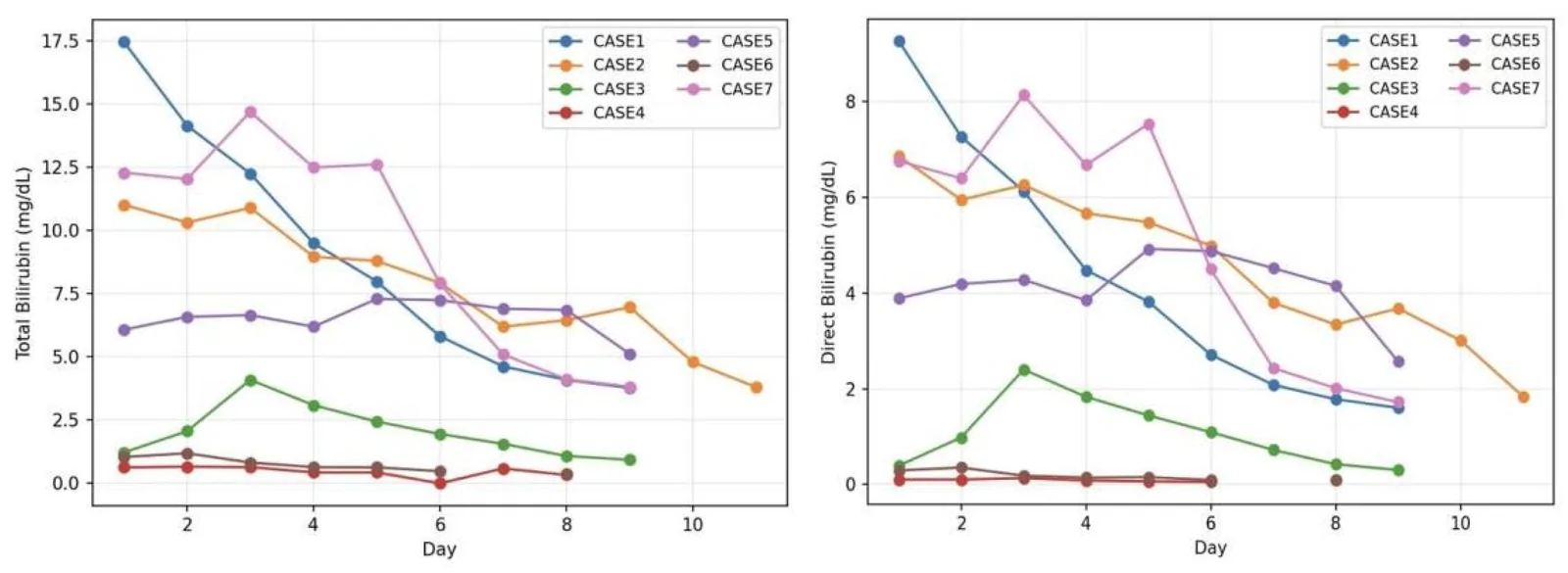
Total bilirubin (**left**) and direct bilirubin (**right**) from admission to discharge in the same patients.

Median total bilirubin was 6.07 mg/dL (IQR 1.13–11.64; range 0.63–17.45). It was above 2 mg/dL in four patients at admission and later in a fifth. Median direct bilirubin was 3.89 mg/dL (range 0.10–9.27), and median indirect bilirubin was 2.18 mg/dL (range 0.53–8.18). Figure 2 shows the course of bilirubin. Median INR was 1.07 (range 0.93–1.70). Albumin and renal function were normal. One patient had a creatine kinase of 910 U/L. Median MELD-Na was 16 (IQR 12–20; range 7–21).

Median LDH at admission was 514 U/L (IQR 395.5–649; range 214–751). It was above the ULN (250 U/L) in six patients and fell during the stay in all of them.

### 3.3. Imaging Findings

**Figure 3:**
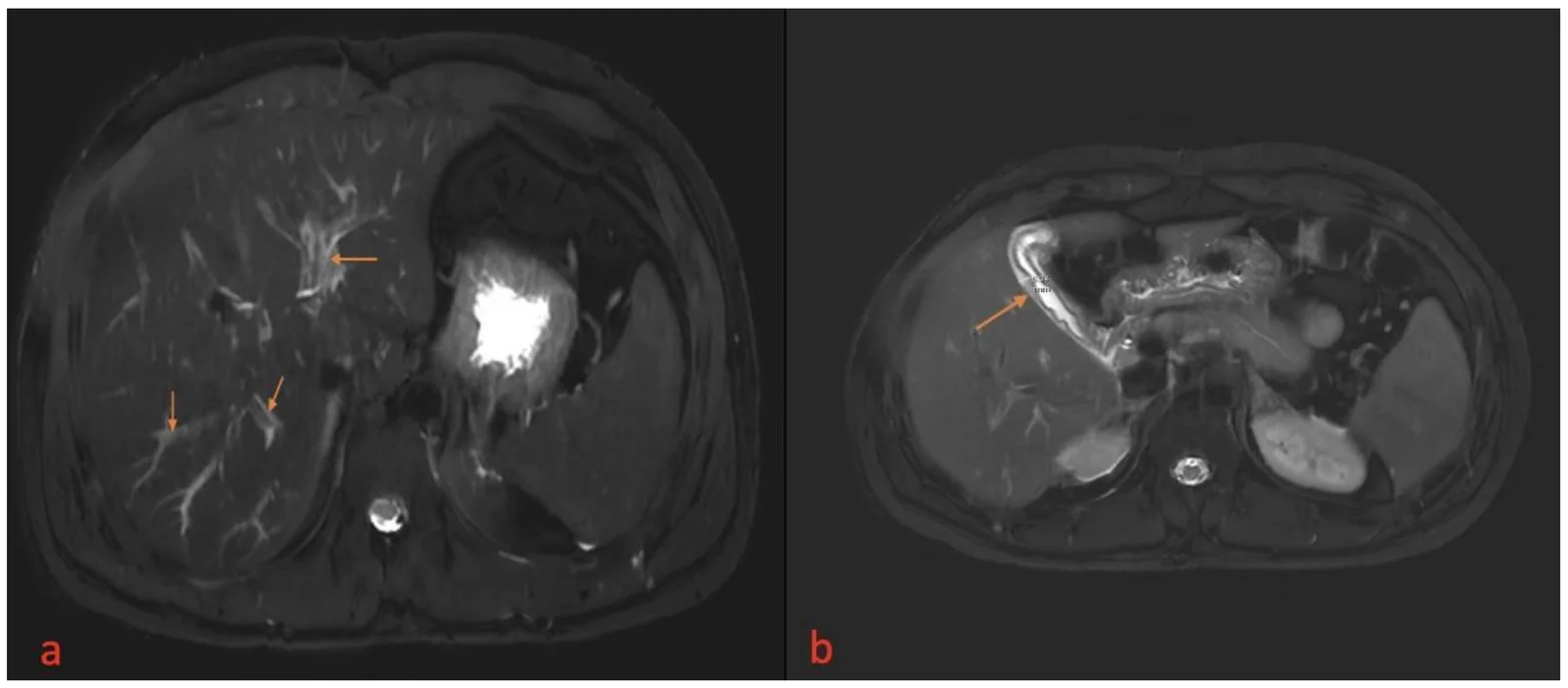
Axial fat-suppressed T2-weighted MR images. (**a**) Periportal edema (arrows). (**b**) Gallbladder wall thickening (arrow).

Ultrasound and CT at admission showed findings of acute hepatitis. Hepatomegaly was present in 3 of 7 patients (43%), periportal edema in 6 (86%; Figure 3a), and gallbladder wall thickening in 3 (43%; Figure 3b). Two patients (29%) had small reactive lymph nodes at the porta hepatis, and two (29%) had heterogeneous liver parenchyma. There was no steatosis, biliary dilatation, vascular abnormality, or ascites. On non-contrast CT, the liver–spleen attenuation difference was normal.

**Figure 4:**
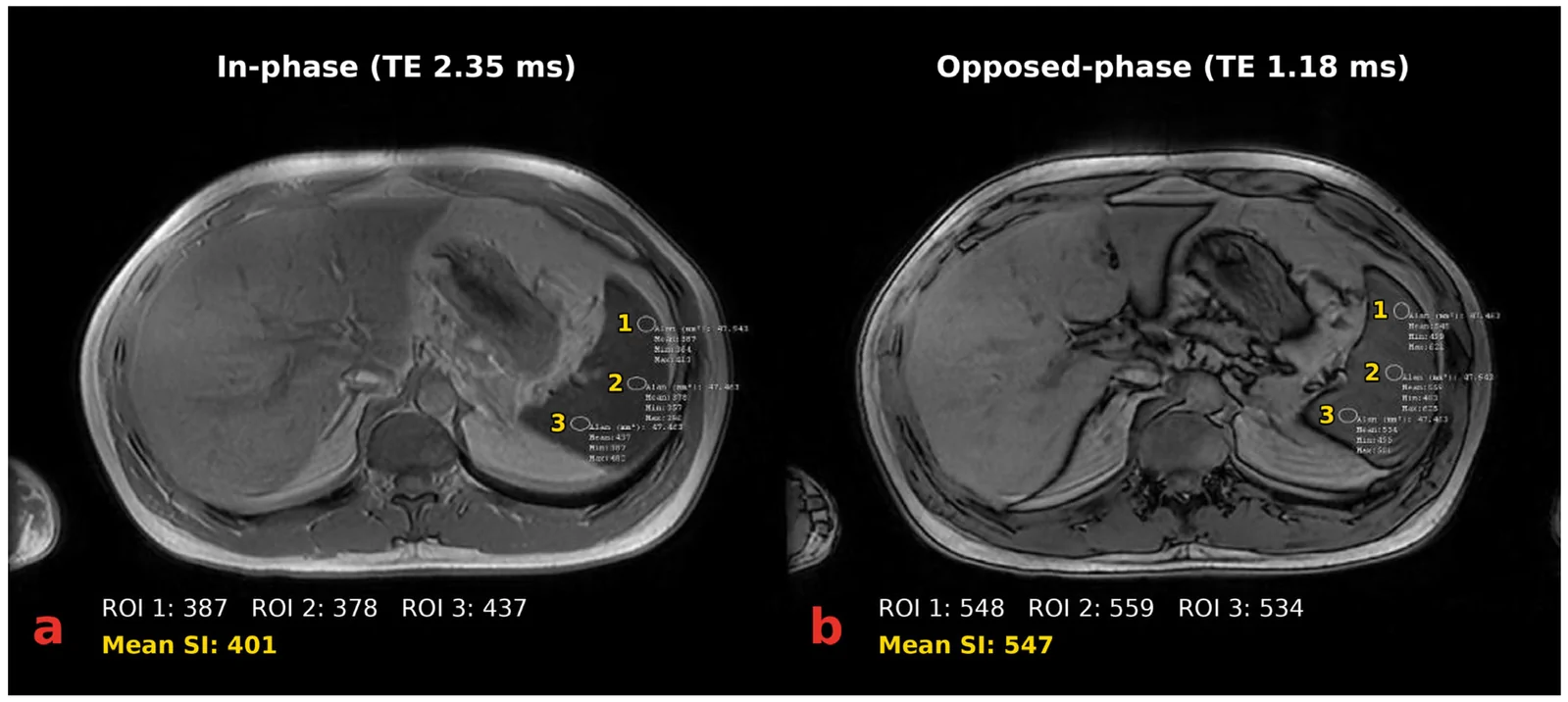
Dual-echo chemical-shift MR images of the spleen in one patient. (**a**) In-phase image. (**b**) Opposed-phase image. The spleen is darker on the in-phase image. The three ROIs and their mean signal values are shown (SIR in this patient ≈ 0.73).

On chemical-shift MRI, the spleen lost signal on the in-phase images in all seven patients (Figure 4). Median splenic SIR was 0.77 (IQR 0.74–0.81; range 0.70–0.87) in patients and 1.05 (IQR 1.02–1.08; range 0.98–1.12) in healthy controls. Every patient had a lower SIR than every control. With the post hoc cut-off of 0.90, 7 of 7 patients and none of the controls had an SIR below 0.90. The liver showed no fat on the opposed-phase images. Table 3 compares the two groups.

### 3.4. Clinical Course

Median hospital stay was 9 days (range 5–11). AST fell below 100 U/L by discharge in six patients. ALT fell more slowly and was still above 100 U/L at discharge in six patients. Pain and nausea resolved, and bilirubin fell in all patients. All patients were discharged in stable condition, and no patient died. Follow-up MRI was not done, and follow-up after discharge is not yet complete.

## 4. Discussion

In this cluster of seven workers with acute hepatitis after petroleum sludge exposure, the spleen was darker on in-phase than on opposed-phase images. The median SIR was 0.77 in patients and 1.05 in healthy controls. To our knowledge, this pattern has not been reported in hydrocarbon-related liver injury. All patients came from one exposure event, so we regard the finding as preliminary.

Even normal tissue loses some signal at the later echo because of T2* decay. In healthy tissue, the ratio is therefore expected to be close to 1; in our controls, the median was 1.05 (range 0.98–1.12). In our sequence, the in-phase echo was the second echo (TE 2.35 ms, compared with 1.18 ms for the opposed-phase echo). An SIR of 0.77 means that the signal fell faster than expected. The usual cause of this in the spleen is iron stored as ferritin or hemosiderin in macrophages, for example after hemolysis or transfusion [15,16,17]. If we assume simple mono-exponential decay, an SIR of 0.77 and an echo time difference of 1.17 ms give an R2* of about 220 s^−1^. For comparison, liver R2* in healthy adults is about 30 s^−1^ at 1.5 T [29], and R2* values at 3 T are about twice those at 1.5 T [19]. This is only a rough estimate. Two echoes are not enough to measure R2* reliably. Multi-echo R2* mapping is the proper method [17,18,19].

Other explanations must be considered. The first is a technical effect. Both echoes are acquired in the same repetition, from the same slice, with the same coil, shim, and receiver settings. Coil sensitivity and receiver gain therefore cancel out in the ratio. Poor shimming or susceptibility effects near air in the stomach, colon, or lung base can still cause more signal loss at the longer echo. We placed the ROIs away from the splenic edges and the gastric fundus. Notably, none of the healthy controls examined using the same MRI protocol demonstrated paradoxical splenic signal loss. We cannot fully exclude a technical effect. The second is motion. The two echoes are acquired about 1 ms apart, so breathing affects both images almost equally. The third is splenic congestion or altered blood flow. More deoxygenated blood in the red pulp can shorten T2*, but at these short echo times the effect should be small. The fourth is the normal difference between red and white pulp. This difference mainly matters on contrast-enhanced images, and we averaged three ROIs. Fat in the spleen would raise the ratio above 1, which is the opposite of our finding. Finally, we cannot exclude a deposit of other paramagnetic material from the sludge, because its content was not measured.

One possible explanation is hemolysis followed by iron uptake in splenic macrophages. Our data do not prove this. LDH was raised at admission in most patients and fell during the stay. Indirect bilirubin was also raised in several patients (median 2.18 mg/dL). These findings fit with mild hemolysis, but they are not specific [30]. Injured liver cells also release LDH, and liver injury alone can raise indirect bilirubin [31]. We did not measure haptoglobin, reticulocyte count, ferritin, serum iron, or transferrin saturation, and we did not examine a blood smear; therefore, we cannot say whether any patient had hemolytic anemia. We had no biopsy and no R2* mapping. The mechanism therefore remains a hypothesis.

Healthy controls show that this signal loss is not a normal finding on our protocol. They cannot show whether the sign comes from the toxin itself or from acute liver inflammation in general. We did not study patients with other forms of hepatitis, with other toxic or drug-induced liver injury, or with hemolysis but no liver disease. We therefore cannot say that the finding is specific to hydrocarbon exposure. The seven patients also shared the same exposure and were scanned in the same period. They are not seven independent observations. The consistency within this cluster is interesting and generates a hypothesis, but it does not exclude chance, a shared technical factor, or another shared characteristic. The patient with only mild liver test changes (case 4) also had the splenic finding. This may point to a link with the exposure rather than with the severity of liver injury, but one patient is not enough to support this.

The other imaging findings were similar to those of acute hepatitis from other causes. Periportal edema was seen in most patients. It appeared as high T2 signal along the portal branches and probably reflects fluid and inflammation in the portal tracts [9,32]. Three patients had diffuse gallbladder wall thickening without stones. This is common in acute hepatitis and is thought to reflect wall edema [9,28,33]. No patient had a focal lesion, biliary obstruction, or vascular thrombosis. In toxic hepatitis, imaging mainly serves to exclude other diseases.

Earlier reports of solvent-related liver injury focused on clinical and laboratory data. In the study of Pérez et al., about half of the refinery workers with raised transaminases had fatty liver on ultrasound [7]. None of our patients had fat in the liver on MRI. The two groups differ in type and length of exposure and in methods, so we cannot say why the findings differ. In the series of Teschke, most patients recovered with treatment [8]. Our patients also recovered with supportive care, including N-acetylcysteine and ursodeoxycholic acid. By the R ratio, the injury in our patients was hepatocellular. Raised GGT and direct bilirubin showed an added cholestatic component. Solvents can cause both hepatocellular injury and intrahepatic cholestasis [3,10].

This study has limitations. First, it is a small retrospective study of one exposure cluster at one center, so the results may not apply to other exposures. One patient had only mild liver test changes, and one admission ALT value was probably recorded incorrectly. Second, one reader measured the images without blinding, and we did not test reproducibility. Third, the SIR cut-off of 0.90 was chosen post hoc and has not been validated. Fourth, we did not do biopsy, R2* mapping, or a full iron and hemolysis panel (haptoglobin, reticulocyte count, blood smear, ferritin, and iron studies). LDH and indirect bilirubin are not specific for hemolysis, so iron deposition was not confirmed. Fifth, we did not do follow-up MRI, so we do not know whether the splenic signal returned to normal. Sixth, the controls were healthy people only, and they were scanned retrospectively. Finally, the content of the sludge was not measured. Future studies should use multi-echo R2* mapping [19], ferritin and iron studies, haptoglobin, reticulocyte count, and a blood smear. They should also include blinded readers with repeat measurements, patients with other liver diseases, and follow-up MRI after recovery.

## 5. Conclusions

In seven workers with acute hepatitis after one petroleum sludge exposure, the spleen had lower signal on in-phase than on opposed-phase MRI. This was not seen in healthy controls. It suggests a T2* effect, possibly from iron deposition after hemolysis, but this is not proven. We regard it as a candidate imaging sign that needs independent validation. At present, it cannot be used to diagnose toxic hepatitis. It may, however, prompt a careful exposure history.

## Figures and Tables

**Table 1:** Clinical and laboratory characteristics of the seven patients at admission.

Variable	Median [IQR] or *n* (%)	Range
Age, years	26 [22.50–27.50]	18–35
Male sex	7 (100%)	—
Hepatomegaly (US/CT/MRI)	3 (43%)	—
Periportal edema (US/MRI)	6 (86%)	—
Parenchymal heterogeneity (CT/MRI)	2 (29%)	—
Porta hepatis/celiac lymph nodes	2 (29%)	—
Gallbladder wall thickening	3 (43%)	—
Hospital stay, days	9 [5–11]	5–11
Albumin, g/L	42.90 [42.60–44.45]	40.1–46.0
AST, U/L	227 [160–315.50]	24–345
ALT, U/L *	481 [351.50–575.00]	36–668
AST/ALT ratio *	0.34 [0.31–0.62]	0.28–1.01
ALP, U/L	128 [109.50–135.00]	48–172
GGT, U/L §	317 [227–509]	25–624
R ratio (n = 6) †	12.7 [11.4–14.7]	10.7–22.4
Total bilirubin, mg/dL	6.07 [1.13–11.64]	0.63–17.45
Direct bilirubin, mg/dL	3.89 [0.34–6.81]	0.10–9.27
Indirect bilirubin, mg/dL	2.18 [0.79–4.83]	0.53–8.18
INR	1.07 [0.98–1.24]	0.93–1.70
PT, s	12.60 [11.60–14.35]	10.9–18.6
Lactate, mmol/L	1.30 [1.12–1.44]	0.9–2.1
BUN, mg/dL	10.20 [9.85–13.29]	7.0–18.0
Creatinine, mg/dL	0.78 [0.70–0.85]	0.62–0.95
CK, U/L	135 [108–309]	62–910
D-dimer, mg/L	0.99 [0.62–1.02]	0.31–2.48
Arterial pH	7.43 [7.39–7.44]	7.35–7.47
LDH, U/L	514 [395.50–649.00]	214–751
MELD-Na	16 [12–20]	7–21
Splenic SIR (in-phase/opposed-phase)	0.77 [0.74–0.81]	0.70–0.87

ALP, alkaline phosphatase; ALT, alanine aminotransferase; AST, aspartate aminotransferase; BUN, blood urea nitrogen; CK, creatine kinase; GGT, gamma-glutamyl transferase; INR, international normalized ratio; IQR, interquartile range; LDH, lactate dehydrogenase; MELD-Na, Model for End-Stage Liver Disease-Sodium; PT, prothrombin time; and SIR, signal intensity ratio. * For case 5, the day-2 ALT was used (see text), and the AST/ALT ratio uses the day-2 AST and ALT. † Calculated for the six patients with raised ALT. § For case 1, the day-2 value was used (day 1 missing).

**Table 2:** MRI acquisition parameters (3.0 T).

Parameter	Dual-Echo T1 GRE	T2 FSE (Fat-Suppressed)	DWI
Plane	Axial	Axial	Axial
TR, ms	111	8000	7058
TE, ms	1.18 (OP)/2.35 (IP)	68.9	69
Flip angle, °	70	90	-
Slice thickness	5 mm	5 mm	5 mm
Slice gap	0.5 mm	0.5 mm	0.75 mm
Field of view	380 × 380 mm	380 × 380 mm	360 × 360 mm
Acquisition matrix	1080 × 1080	1080 × 1080	1076 × 1076
Breathing	Breath-hold	Respiratory-triggered	Respiratory-triggered
Acquisition time	15–20 s	3–5 min	3–5 min

DWI, diffusion-weighted imaging; FSE, fast spin-echo; GRE, gradient-echo; IP, in-phase; OP, opposed-phase; TE, echo time; and TR, repetition time.

**Table 3:** Comparison of patients and healthy controls.

Variable	Patients (*n* = 7)	Healthy Controls (*n* = 15)	*p*
Age, years	26 [22.5–27.5]	26 [22–28]	-
Male sex	7 (100%)	15 (100%)	-
Splenic SIR	0.77 [0.74–0.81]	1.05 [1.02–1.08]	<0.001
Splenic SIR, range	0.70–0.87	0.98–1.12	-
SIR < 0.90 (post hoc cut-off)	7 (100%)	0	-

Values are median [IQR] or *n* (%). Splenic SIR was compared with the two-sided exact Mann–Whitney U test. Age and sex were not tested because the groups were matched by design. SIR, signal intensity ratio.

## Data Availability

The data are available from the corresponding author on reasonable request. They are not public because of patient privacy.
